# Supplementary material for: Microstructure and Mechanical Properties of Dual Scaled NbC/Ti2AlC Reinforced Titanium–Aluminum Composite
Source: Materials (Basel). 2023 Jun 28;16(13):4661. doi: 10.3390/ma16134661 (PMC10342962; doi:10.3390/ma16134661)
Supplement: Supplementary file 1 [file materials-16-04661-s001.zip › materials-2441207-supplementary.pdf]

materials

MDPI

Supplementary Files

Table S1. Parallel test data of room temperature compression tests (group 1).

Properties \ Samples	TiAlNb	T0.5	T1	T2	T3
Compression Strain (%)	18.4	23.2	24.1	22.7	19.9
Compression Stress (MPa)	1538	2020	2301	2206	2038
Fracture toughness (MPa/m^2)	19.0	23.9	25.9	25.1	21.7

Table S2. Parallel test data of room temperature compression tests (group 2).

Properties \ Samples	TiAlNb	T0.5	T1	T2	T3
Compression Strain (%)	17.0	22.1	24.9	21.5	18.0
Compression Stress (MPa)	1570	2069	2240	2224	2105
Fracture toughness (MPa/m^2)	15.9	23.2	27.2	25.9	20.7

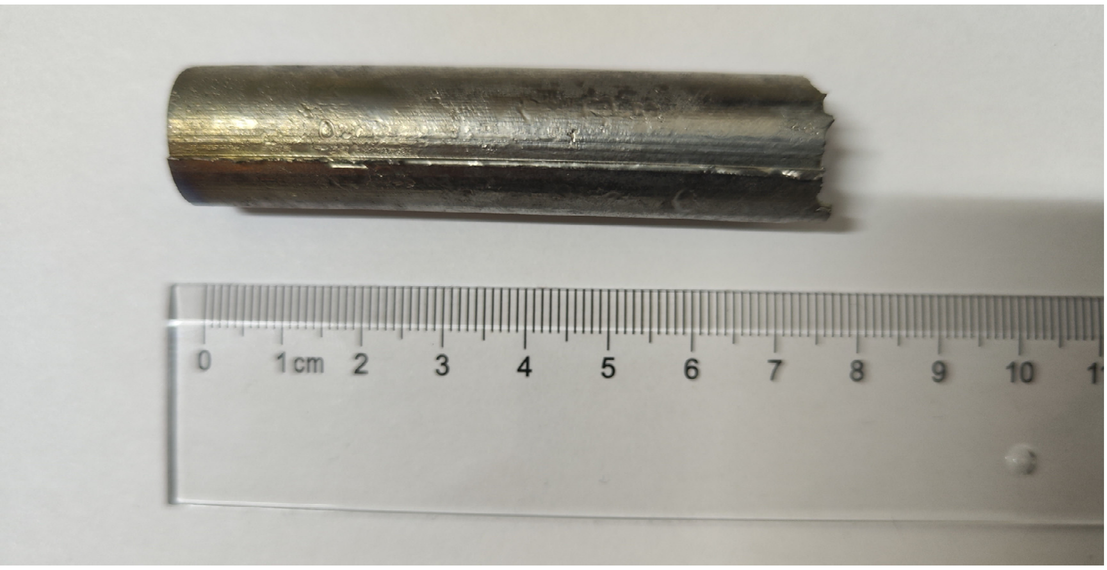

Figure S1. Macro photo of the sample T1.
